# Distribution Transformer Parameters Detection Based on Low-Frequency Noise, Machine Learning Methods, and Evolutionary Algorithm

**DOI:** 10.3390/s20154332

**Published:** 2020-08-04

**Authors:** Daniel Jancarczyk, Marcin Bernaś, Tomasz Boczar

**Affiliations:** 1 Department of Computer Science and Automatics, University of Bielsko-Biala, 43-309 Bielsko-Biala, Poland; mbernas@ath.bielsko.pl; 2 Institute of Electric Power Engineering and Renewable Energy, Opole University of Technology, 45-758 Opole, Poland; t.boczar@po.opole.pl

**Keywords:** low-frequency sensor, power transformer, machine learning, low-frequency noise, genetic algorithm

## Abstract

The paper proposes a method of automatic detection of parameters of a distribution transformer (model, type, and power) from a distance, based on its low-frequency noise spectra. The spectra are registered by sensors and processed by a method based on evolutionary algorithms and machine learning. The method, as input data, uses the frequency spectra of sound pressure levels generated during operation by transformers in the real environment. The model also uses the background characteristic to take under consideration the changing working conditions of the transformers. The method searches for frequency intervals and its resolution using both a classic genetic algorithm and particle swarm optimization. The interval selection was verified using five state-of-the-art machine learning algorithms. The research was conducted on 16 different distribution transformers. As a result, a method was proposed that allows the detection of a specific transformer model, its type, and its power with an accuracy greater than 84%, 99%, and 87%, respectively. The proposed optimization process using the genetic algorithm increased the accuracy by up to 5%, at the same time reducing the input data set significantly (from 80% up to 98%). The machine learning algorithms were selected, which were proven efficient for this task.

## 1. Introduction

The transformer is a passive electrical device that transfers electrical energy from one electrical circuit to another, or multiple circuits. The transformer is composed of the main parts: The primary and secondary winding wound around the same core and the air or oil cooling system. Noise emitted by the transformer is a vibrio-acoustics problem. The acoustic vibrations of a transformer can be generated by the following main phenomena [1,2]:

- Coil vibrations depending on the current amplitude and winding clamping compression;
- Core vibration depending on magnetostriction or loosening of core clamping;
- Air circulation caused by fans; and
- Work of the pumps circulating the insulation oil.

**Figure 1:**
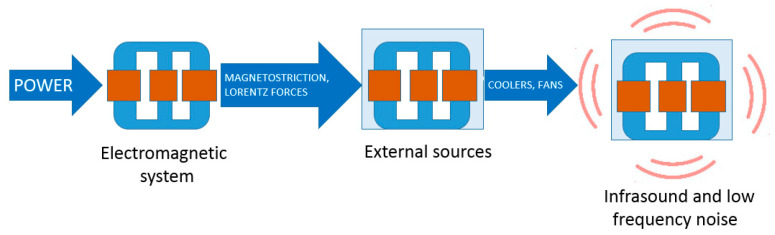
The process of the generation of noise from the vibration of a transformer.

The process of the generation of noise from the vibration of a transformer is shown in Figure 1.

In transformers, the load noise is predominantly produced by axial and radial vibration of the windings. Load noise can also be caused by vibrations in the transformer tank walls and magnetic shields due to the electromagnetic forces produced by the load currents. These electromagnetic forces are proportional to the square of the load currents. The frequency of load noise is usually twice the power frequency. In some cases, the natural mechanical frequency of winding clamping systems may tend to resonate with electromagnetic forces, thereby severely intensifying the load noise. Transformer cores are constructed by stacking layers of thin iron laminations, separated from its neighbors by a thin non-conducting layer of insulation. When the core becomes magnetized, the magnetic field acts between the adjacent plates, stretching and squeezing the adhesive and insulation between them. A transformer is magnetically excited by an alternating voltage and current so that it becomes extended and contracted twice during a full cycle of magnetization. This change in dimension is independent of the direction of magnetic flux, occurring at twice the line frequency. The main source of heat generation in transformers is caused by copper loss in the windings and core. This heat is often removed by cooling fans, which blow air over radiators or coolers. Noise produced by cooling fans usually contribute more to the total noise for transformers of a smaller rating and for low-induction transformers. Cooling equipment noise typically dominates the very low- and very high-frequency ends of the sound spectrum, whereas the core noise dominates in the intermediate range of frequencies between 100 and 600 Hz [3,4,5,6,7].

**Figure 2:**
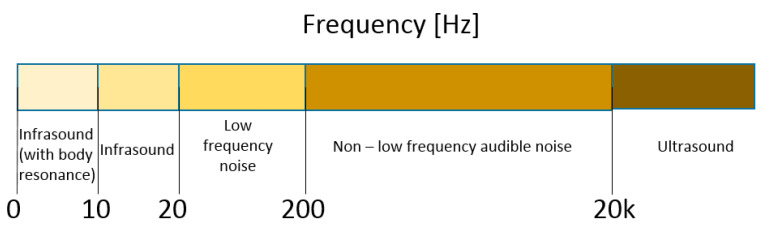
The frequency spectrum of sound and its nomenclature.

For a person with normal hearing, the human hearing range starts low at about 20 Hz. On the other side of the human hearing range, the highest possible frequency heard without discomfort is 20 kHz. Ultrasound is sound waves with frequencies higher than the upper audible limit of human hearing (above 20 kHz). In this description, we focus on the lower end of the frequency spectrum. We are interested in infrasound and low-frequency noise; see Figure 2. The range of analyzed frequencies is up to 200 Hz, taking under consideration body resonance infrasound.

The acoustic emission depends on the anomalies, age, and rated power of the machine. For this reason, the analysis of the generated sound, especially within the spectrum of low-frequency noise, can be useful in determining the rated parameters of the transformer and its diagnostic parameters. It is worth noting that the measurement of low-frequency signals is carried out non-invasively and during normal operation of the distribution transformer.

Our research was extended to remove drawbacks of the previous method [8], and to develop an automatic method for determining the technical parameters and diagnostics of distribution transformers based on the analysis of the characteristics of their low-frequency signals. Using low-frequency signal allows measurement of the device from a distance (50 m). This research presents a method for detecting the base parameters of distribution transformers (type and rated power).

## 2. State-Of-The-Art

Transformers can vary, from a miniature high-frequency audio transformer to a large power transformer, but the operating parameters are the same. These can be divided into eight groups and are posted on the nameplate of any transformer of significant size: Nominal apparent power (VA rating), cooling, transformer rating frequency, voltage, phase, connections, and taps [9]. Based on the low-frequency characteristics of the transformer, we are able to distinguish between the transformer’s operating status, its cooling type, and its apparent power.

In our first study [10], we tried to ascertain the emission level of low-frequency signals generated by distribution transformers at rated conditions. The medium-voltage devices (indoor and overhead type and variety apparent powers) were under study.

The results of the study showed that distribution transformers are a source of infrasound and low-frequency signals. The results demonstrated the similarity in the shape of waveforms of averaged amplitude spectra and time-frequency changes. The waveforms are characterized by relatively dynamic (exponentially) decreasing values of registered sound pressure, which occurs with an increase in the frequency in the range from 10 to 100 Hz [10,11].

Research of a similar nature was conducted for the problem of noise from electrical infrastructure. Piana et al. [12] claimed that the low-frequency disturbance occurs for the tested transformer at two harmonics of for each of two frequencies. Other scientists proposed prognostic and system health management (PHM) for power transformer fault diagnosis. Potential uses for PHM is a condition-based maintenance. This system presents opportunities for the detection of mechanical failures, or for system life cycle management. Li et al. [13] presented the study of a power transformer fault diagnosis using a machine learning-based method with a neural network model. The proposed method uses dissolved gas analysis (DGA) as input data. A frequently used method in the diagnosis of oil-filled power transformers is a partial discharge (PD) detection using an acoustic emission (AE) technique. Many cases of power transformer breakdowns are related to insulation system failures, which might have been caused by the high activity of partial discharges [14]. Kunicki et al. [15] proposed a method for detecting defects of power transformers. This method is based on machine learning classification of selected faults. In this case, input data is AE measurement. The classification process consists of two parts: The first part checks whether the source of the emitted signal is partial discharge or another AE source while the second part allows the identification of the specific AE source type.

The aforementioned research is focused on the life cycle of a distribution transformer measured in an isolated environment, where the external noise influence is minimalized. The classification is based on various machine learning algorithms (ML), which finds a pattern in data based on expert feedback. Therefore, in [12,13,14,15], the supervised learning [16] was used to train a model for various applications. However, in this research, the analyzed data were gathered from a significant distance, thus unsupervised learning was used [17] to find anomaly in the background noise, the source of which was unknown. In the case of maintenance operation issues [14], reinforcement learning [18] was used to find optimum actions for a given operation status of a transformer. In this paper, the proposed method utilizes both supervised and unsupervised learning. The supervised learning is used to build a classification model based on evolution strategy and state-of-the-art ML algorithms: k nearest neighbors (kNN) [19], naive Bayes classification (Bayes) [20], support vector machine (SVM) [21,22], random forests [23,24], and neural networks [25,26,27,28]. The unsupervised learning is applied to tackle the background noise.

The accuracy of the constructed model (using the ML algorithm) depends not only on the algorithm but also the type of input data and its representation. Thus, many models use a preprocessing method, which transfers data into a new variable space [17]. The transformation can be a simple operation as scale transformation (e.g., to the dB scale) or a more complex one, when the nature of data changes [29]. The principal component analysis [29,30,31,32] and canonical correlation analysis [33,34] are commonly used methods for TS. 

The neural networks (especially deep ones), due to the variety of their structures, are both classification and pattern recognition tools [25,26]. Using multiple hidden layers allows the creation of linear and non-linear models. A drawback of this approach is that the training procedure requires a large amount of data and computation power to obtain a high model accuracy. Moreover, finding the optimum set of weights in the case of a multiple hidden layer structure is an NP-complete problem [35]. Thus, a substitute for classic multiple layer perceptron networks was proposed (deep neural network), in which the network is divided into layers with specific functions [28,32]. Significant results using deep neural networks have led them to be the most commonly employed classifiers in machine learning [35,36].

The proposed method is based on two sources of data (transformer sound and background sound gathered in various locations). Thus, these two sources of the sound are considered as independent ones. However, each registered series of sound according to [17,37] is characterized by significant sequential correlations and should be represented as temporal features. There are several methods fitting for this type of data: Hidden Markov [38], sliding window [39], Kalman filter [40], random fields [41], recurrent neural networks [42], and the Welch method. An extended analysis of the methods can be found in [43,44]. In this study, which is an extension of [8], the same Welch method [45] was used to retain the consistency of results. Furthermore, this method was used with success for noise analysis in [11,12].

The previous study [8] proposed a method to automatically detect a known working transformer in close vicinity (50 m). Low-frequency noise generated by transformers (two indoor and two overhead ones) was registered by a dedicated sensor from a distance of 50 m and then classified using the proposed machine learning method. It is worth noting that the research was performed in a real environment. The method used an exhaustive search and Bayesian optimization to find a frequency interval that gave the best detection results. The drawback of the previous method [8] was that only one interval could be searched at a time, while the results showed that depending on the method, various intervals were selected near the following frequencies: 2, 50, and 100 Hz. Despite the drawbacks of the method, a 99% accuracy was obtained for the state of the transformers (on/off) using the random forest, KNN, and naïve Bayes methods.

## 3. Proposed Method

**Figure 3:**
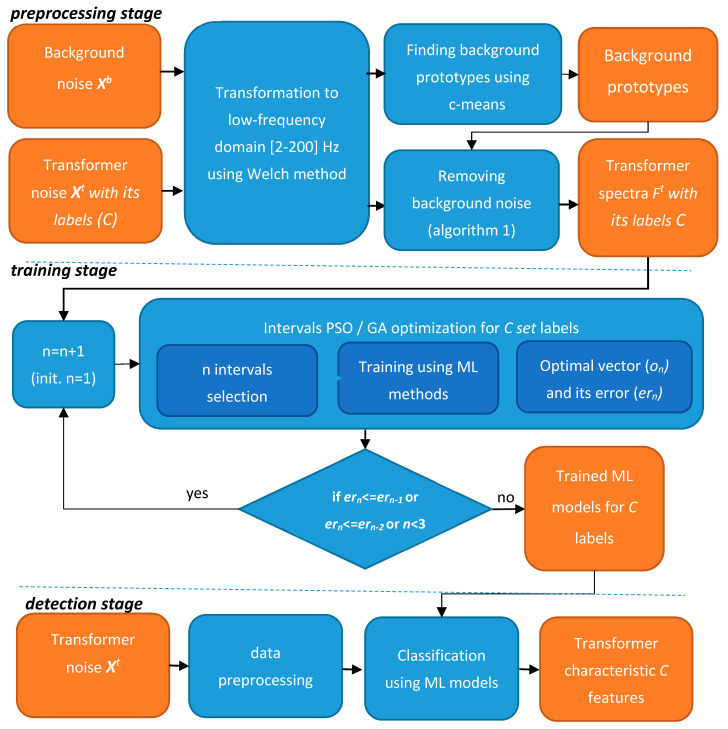
Proposed method for the detection of transformer parameters.

In the research for this paper, the frequency was extended to 200 Hz as it was proven that most information was stored close to 100 Hz and a higher frequency was not examined. Furthermore, the research was performed for one interval at a time while several harmonics were found (1, 50, and 100 Hz). To provide sounder results, the transformer database was significantly extended (from 4 to 16) and several of its parameters were researched. Based on the initial observation and previous research [8], a method was proposed, which finds the optimum frequency representation for distribution transformer features: Model, type, and apparent power. The overview of the method, with a background profile analysis, is presented in Figure 3.

### 3.1. Preprocessing Stage

The input data of the method is a sound registered as a time series (*X*) by a dedicated sensor node. The sensor measures the transformer noise with background noise (*X^t^*) or only background noise (*X^b^*) if the transformer is not present in the vicinity or it is turned off. Each registered time series *X* is a sequence of sound pressure values *x_i_*, where *i* defines its order. 

**Figure 4:**
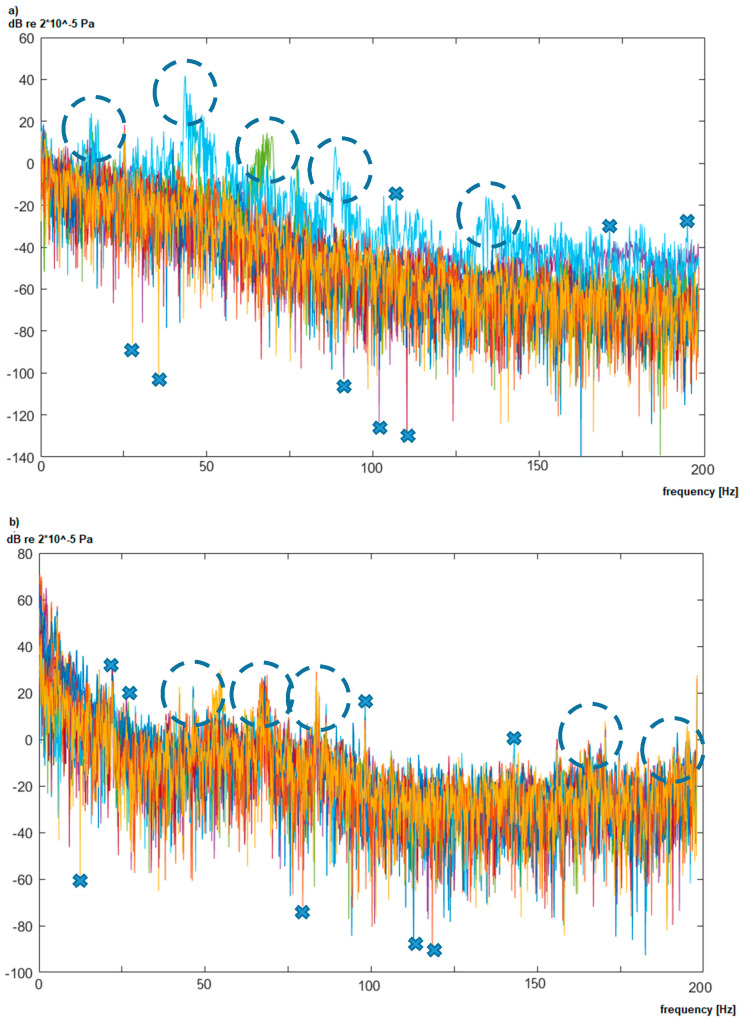
The example of spectra registered in the vicinity of: (**a**) transformer air-type, and (**b**) interior type.

The samples, registered for each distribution transformer (*X^t^*), are converted using the Welch method [8,42] to obtain its frequency representation ($F_{x}^{t}$). The obtained spectra $F_{x}^{t}$ = [*f*_1_, *f*_2_, *…*, *f_m_*] represent the low-frequency spectrum in the range from 2 to 200 Hz with the maximum considered resolution *df* equal to 0.125 Hz (thus *m* = 1585). The *df* value lower than 0.125 Hz would require a significant recording time (over 10 s a sample) and as it was proven in this research that the higher resolution did not increase the classification accuracy. The spectra samples are represented as ***F^t^*** set (***F****^t^* = {$F_{x}^{t},\;x=1,\;\ldots ,\;tmax$}, where *tmax* is a number of samples). Each vector $F_{x}^{t}$ is described by basic transformer parameters (*C* = {*c*_1_,*c*_2_,*c*_3_}), i.e., transformer model (*c*_1_), its type (*c*_2_), and apparent power value (*c*_3_). The characteristic spectra registered in the vicinity of two transformers are presented in Figure 4. 

**Figure 5:**
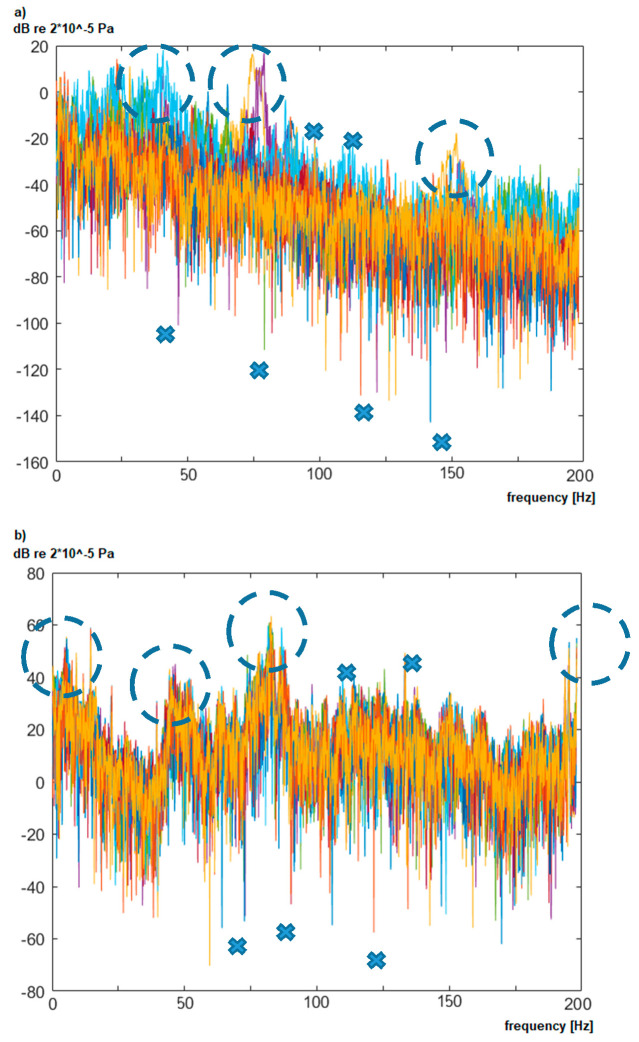
Noise background for various positions: (**a**) outdoor background, (**b**) indoor background.

Each plot in Figure 4a,b presents measurements of the noise characteristic in the vicinity of the same transformer. Measured values for the specific frequency (marked with dashed circles), single peaks (marked with crosses), as well as changes of the amplitude within the whole spectrum can vary significantly. This is caused by the measured distance of 50 m, where other sources of noise are registered as well. Moreover, the transformer load, as presented in [6], can also influence the value of noise, especially in the 50 Hz area. Therefore, an additional source of data was used to identify the anomalies, which originate from analyzed transformers. The background noise in multiple localizations was registered (denoted as $F_{x}^{b},\;x=1,\;\ldots ,\;bmax$), where the noise is not biased by a transformer noise. The registered background characterizes interiors, fields, forests, and the home environment. An example of the background is presented in Figure 5.

The analyzed background noise (without transformers in vicinity) shows that its characteristic are not constant and also change in time. It is characterized by the same type of variations; however, it is registered more frequently for specific frequencies (e.g., 75 Hz). In case of indoor background, registered in Figure 5b, additional noise was registered for the 50 Hz harmonics and near 10 Hz. This noise is generated by electric devices, which are part of the production areas. It is nearly impossible to gather background characteristic for each transformer, because they are part of the energy infrastructure and they cannot be turned off easily. Therefore, the gathered backgrounds (Figure 5) are characteristic for a specific area, not for a specific transformer. Moreover, as it is shown in Figure 4 and Figure 5, the background changes with time due to temporal occurrences like the influence of constructions, large objects, or vehicles.

Initial work was conducted to find a universal background using the following estimates: Mean, median average, and maximum and minimum value; however, this approach generated worse results. Therefore, based on the analysis, we propose to define several background characteristics and adopt them for each spectrum based on the similarity measure. The fuzzy c-means algorithm [46] was used to find characteristic backgrounds and then, based on the similarity to the analyzed spectra, the appropriate one was selected. To select the background ***F^b^*** for transformer spectra $F_{x}^{t}$, the following procedure (Algorithm 1) is given:


**Algorithm 1**

V = cmean(***F^b^***,*vmax*), where: cmean-fuzzy c-mean function [42], vmax-number of prototypes,mt = mean($F_{x}^{t}$);mxw = 0; minVal = INF; minid = −1;for j = 1:vmaxmx = mean(V(j)));val1 = mean((($F_{x}^{t}$−mt)−V(j)−mx).^2.^0.5);val2 = mean($F_{x}^{t}$)−mean(V(j));if (val1 < minVal && val2 > 0)minVal = val1;minid = j;mxw = mx;endendresult = $F_{x}^{t}$−V(minid).


**Figure 6:**
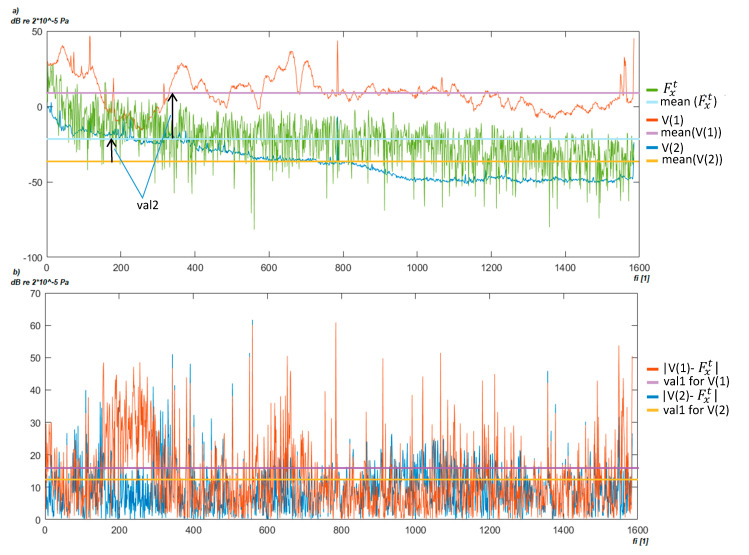
Example of the c-means background cluster fitted to a transformer: (**a**) calculate val2 measure, (**b**) calculate val1 measure.

Algorithm 1 generates a set of characteristic backgrounds denoted as V based on background data (line 1). Then, the most similar background noise V(j) is searched for in the analyzed $F_{x}^{t}$ spectrum (lines 4–10). The V spectra are compared based on similarities for the same frequencies (*val1*) as well as its average value (*val2*). Figure 6 presents the similarity calculation process. At first, the average value for each potential background is calculated as presented in Figure 6a. The difference between average values (denoted as val2) is defined if the background noise is stronger than the registered transformer noise. Only those prototypes V(j) are considered, in which the average noise is lower than the analyzed spectrum, $F_{x}^{t}.$ Next, the potential background spectra shapes are compared according to formula in line 6. The result of the subtraction is presented in Figure 6b. The obtained mean value of this substation is treated as a similarity measure. In the example presented in Figure 6, the V(1) prototype will not be selected because its mean noise level is higher (val2 = −50) and its similarity measure (val1) is lower than in the case of V(2). Thus, the V(2) prototype is selected as the $F_{x}^{t}$ background. The background is subtracted from the spectrum (line 14). The operation is performed for every spectrum ***F****^t^* = {$F_{x}^{t},\;x=1,\;\ldots ,\;tmax\}$. Then, the input set *F^t^* with the subtracted background is used to find the optimum frequency interval representation in the next step. 

### 3.2. Training Stage Using ML and Evolutionary Algorithms

The search for optimum data representation (frequency space and its resolution) is performed using the evolutionary algorithm. Two state-of-the-art evolution methods, genetic algorithm (ga) and particle swarm optimization (PSO), were applied for each of the C features separately.

The first method, due to the discrete search space, was based on integer programming. In this implementation, special creation, crossover, and mutation functions cause variables to be integers [47]. The implemented genetic algorithm attempts to minimize a penalty function, which is combined with binary tournament selection to select individuals for subsequent generations [48].

The PSO algorithm is based on the implementation proposed by Kennedy and Eberhart [49], using modifications suggested in Mezura-Montes and Coello Coello [50] and in Pedersen [51]. In contradiction to the ga algorithm, it creates the initial particles, and assigns initial velocities to them. Then, it evaluates the objective function at each particle location and determines the best (lowest) function value and the best location. Finally, it chooses new velocities, based on the current velocity, the particles’ individual best locations, and the best locations of their neighbors. Initially, the particles are uniformly distributed within bounds.

Both methods use a similar population/swarm size parameter equal to 200 as well as a total error function equal to 1 × 10^−4^.

The searched frequency space is described as an ordered set of frequency intervals described by a *t_i_* tuple. The initial number of intervals *n* is equal to 1 and its value increases with each iteration. Each tuple *t_i_* = [*l_i_*, *h_i_*, *s_i_*], *i* = 1, *…*, n, where: *l_i_* = [2, 200], *h_i_* = [2, 200], *l_i_ < h_i_*, and *h_i_ < l_i+_*_1_ define respectively the lower (*l_i_*) and upper (*h_i_*) frequency bound and $s_{i}\in N$—defines the sample resolution for the given tuple. The described constraint allows limitation of the searching space to a considered frequency interval [2, 200] and ensures that intervals will not overlap. 

Using evolution algorithms, a population of potential vectors *o_n_* = {*t_i_*, *i* = 1, *…*, n} is selected. With each generation, the *o_n_* is selected, which has the lowest error ratio calculated as the misclassification accuracy using cross-validation results. Additionally, the secondary aim of optimization is to find the minimal set of frequency, which did not influence the result significantly. Thus, the following fitness function (*ff*) was proposed:

(1) $$ff\left(F^{t},o_{n}\right)=\min(cv\left(ML\left(F^{t},o_{n}\right)\right)+\frac{\sum_{i=1}^{n}[\frac{h_{i}-l_{i}+1}{s_{i}}]}{1000000}$$

where ML is a function performing training for data and cv-return cross-validation error in the range [0, 1].

Using this approach, we ensure that the result with the highest accuracy will be selected, while the results with less samples will be favored in case of a comparable accuracy level. Taking under consideration the maximum number of samples, the value fitness function will be changed by 0.0015.

Several optimization algorithms as well as ML methods were analyzed to find an optimum solution. In this section, all used algorithms will be described.

The M model is a result of the selected ML method and data provided as a *F^t^* vector and interval set *o_n_*. The following methods were researched to find the optimum classifier:

- kNN model, where class is determined by the *k* closest vectors in a defined frequency space. As a measure of distance, the classic Euclidean distance was used with an initial *k* value equal to 5;
- Bayes approach, where a family of probabilistic classifiers was applied according to [20], thus no additional parameters were needed. It was assumed that each frequency characteristic is independent (Bayes theorem);
- Multivariable support vector machines, where binary learners were used to train the characteristics of each transformer and its parameters. In contrast to the radial basis function 45 applied in [8], in this research, multi-linear SVM was used, which finds a hyperplane that is a linear function of each input feature and the rest of the features. The implementation was adopted from [18,19,52];
- Random forest, where 10 trees create a forest, was used make the method more robust [23]. The result of a class is determined by voting. The parameter value was selected empirically;
- Neural network, where the multilayer perceptron network (MLP) was selected as the architecture. The MLP was selected to reduce the computation complexity of each iteration for the evolution algorithm. Several architectures were researched. Finally, 2 hidden layers and 20 neurons per layer were used to take the non-linear characteristic of the analyzed data into consideration [26].

### 3.3. Detection Stage

The detection stage is used to verify a proposed model and can also be applied for detecting transformer parameters *C* in the considered area. The registered noise is preprocessed in a similar way as data in the preprocessing stage. The sample is transformed to the frequency domain using the Walsh method and algorithm 1 is performed. However, in this case, the generated prototypes in the first stage are used. The preprocessed data are then processed in parallel by three ML models generated for each of three C features. As the result, the basic transformer parameters are determined.

## 4. Results and Discussion

**Figure 7:**
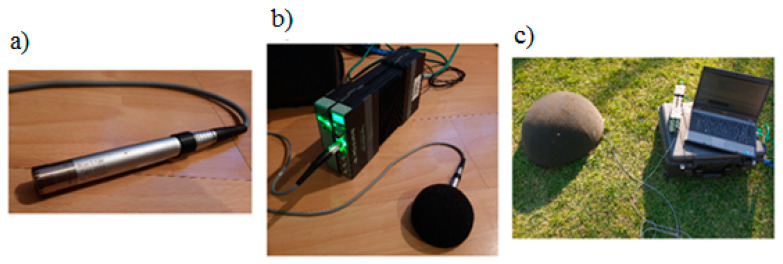
Used measurement system from Brüel & Kjær: (**a**) microphone, (**b**) digital meter, and (**c**) complete system.

Measurements were made using specialized equipment from Brüel & Kjær (company name details: Brüel & Kjær Sound & Vibration Measurement A/S, DK-2850 Nærum, Denmark). The system consists of a ½-inch free-field microphone type 4190, a preamplifier type 2669 L, and a digital signal meter with registration function LAN-XI type 3050-A-60 from Brüel & Kjær. The connected system and its block diagram are shown in Figure 7.

The microphone was designed for very precise measurements in the free field. Its lower cut-off frequency is 1.2 Hz and the transmission characteristic is linear within ±3 dB from 1.2 Hz to 20 kHz. This microphone is characterized by high sensitivity (50 mV/Pa) and a dynamic range from 15 to 148 dB. The lower range of the measured frequencies can be defined by the user using high-pass filters (0.7 or 7 Hz).

The system was managed from the personal computer using PULSE LabShop application version 15.1.0. This is dedicated software that defines all operating parameters, records the measured signals, and preprocesses and visualizes them after measurements in offline mode.

The measurements were taken in a continual process over several hours, with a sampling frequency of 51.2 kHz (sampling for the whole human hearing range). Additionally, all measurements were carried out far from major roads and motorways, which are considered to be sources of low-frequency noise. The minimal sample length was defined by the Welch window size and it was defined as 10 s.

The research was conducted using 16 distribution transformers and 6 backgrounds for various areas and types (indoor and overhead, dry-type, and oil-type transformers and their apparent power in the range of 100–2500 kVA). All tested transformers reduce the voltage from 15 to 0.4 kV with a mains frequency 50 Hz. A detailed description of the tested transformers is provided in Table 1.

Initial work was performed to find an optimum background representation. It was achieved by finding a set of background prototypes giving the best results in terms of classification. The classification considered the following basic transformer parameters: Specific transformer model (*c*_1_), transformer type (indoor/outdoor) (*c*_2_), and its apparent power (*c*_3_). The results are presented in Table 2. The values were obtained using the kNN classifier.

**Figure 8:**
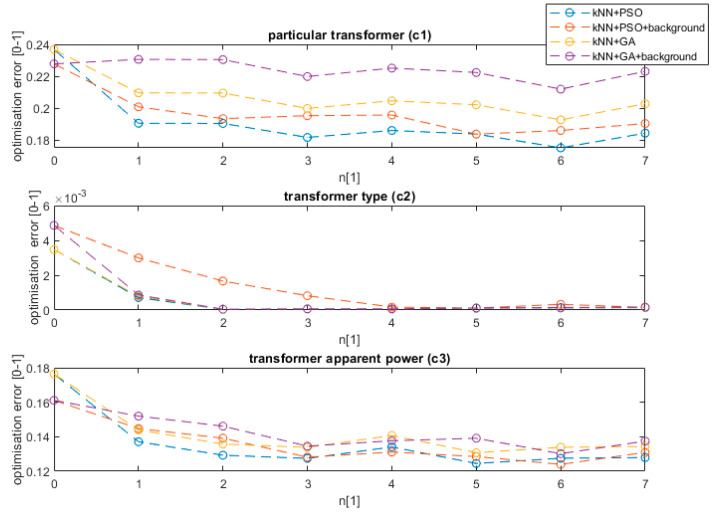
The training results for the optimization algorithm number of intervals n and feature *c*_1_, *c*_2_, and *c*_3_.

The results show that the background selection influences *c*_3_ more than *c*_1_ and *c*_2_. This is caused by the fact that background noise has a bigger impact on the magnitude of low-frequency noise registered and can influence the classification. The initial results confirmed that the characteristics of the background noise for air transformers and indoor transformers are significantly different and allow a decrease of the error by more than 1% even at the preprocessing stage. The indoor transformers are usually installed in urbanized areas, thus more sources of noise are present. Then, the proposed method was applied for the kNN classifier with both the pso and ga training methods. Additionally, the variant with and without background subtraction (application of Algorithm 1) was tested. The result of method training is presented in Figure 8.

**Figure 9:**
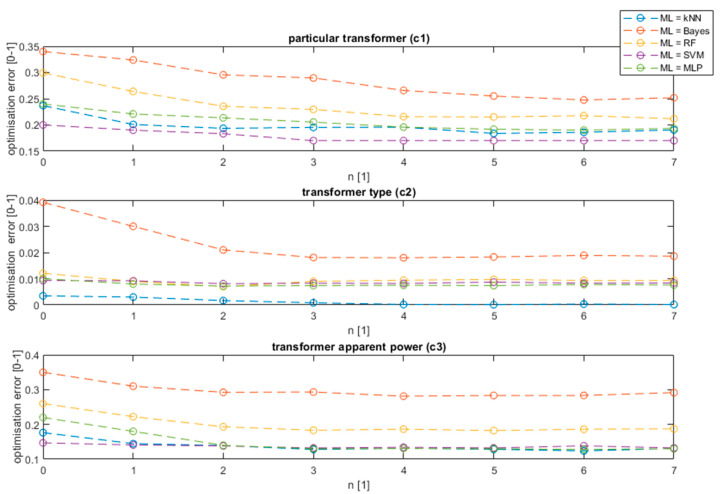
Training for all ML algorithms and analyzed features.

The value n equal to 0 represents the value without interval optimization. The process of finding the optimum interval using the evolution strategy, from the first interval (n equal to 1) reduces the method error on average by 4% in the case of detecting a model of a transformer, five times in the case of the transformer type, and by 5% in the case of the transformer apparent power value. The further increase of the number of intervals steadily decreases the error up to n equal to 3. The best results were achieved for n equal to 6. Above this threshold, the method is over trained and its effectiveness starts to fall. The results confirmed that the used PSO method outperforms the tested ga algorithm by 2% on average for all analyzed features. Additionally, the background data decreases the error in the case of feature *c*_3_. Further analysis showed that the optimization of the data using the background gives a slightly worse result (0.5%) than raw data in the case of *c*_1_ feature. The difference is caused by the characteristic background of a specific area and the tendency to classify the transformer model based on the area background and not transformer noise. Similar training was performed for the remaining ML algorithms. The comparative result for the proposed method is presented in Figure 9 for the PSO algorithm and ML methods as shown in the legend.

**Figure 10:**
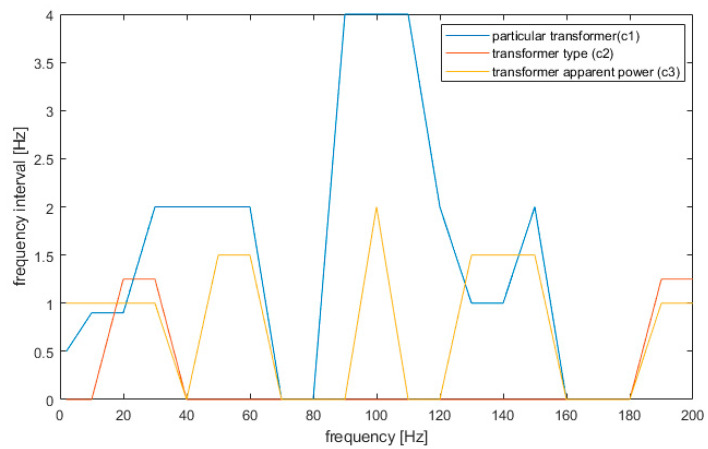
The data usefulness for classification and detection.

The proposed algorithm of interval selection with background subtraction decreased the error in the case of all analyzed ML models. The method was effective in the case of all features; however, the biggest optimization (in percent scale) was noticed in the case of transformer model detection (*c*_1_) and transformer apparent power detection (*c*_3_). In the case of the type of transformer detection, the detection error was close to zero, thus a significant improvement was noticed only in the case of the Bayes classifier. The decreased error can be noticed at the first interval (n = 1), which supports the results obtained in previous research [8], but further decreases can be observed with the interval number increase, obtaining the optimum value at n = 5. This value allows optimal detection of all harmonics of 50 Hz generated by a transformer, and the infrasound interval, which proved to be vital frequencies for transformer classification. The biggest decrease in the error rate can be noticed in case of simple classifiers, such as kNN, Bayes, or random forest. On the other hand, in the case of a complex non-linear SVM model, the decrease is equal to 2% on average. The proposed model not only allows an increase of the detection accuracy but also allows a decrease of the data usage by selecting intervals and the resolution at which the data are vital for classification. The research shows that various ML models require various data sizes; nevertheless, some frequencies’ intervals are characteristic for all classifiers and were repeated in all models. Their ranges are presented in Figure 10.

The analysis definitely shows that in the case of transformer model detection, nearly all frequencies are vital to improve detection. Only frequencies near 70 and 170 Hz contain less information. The results confirm the observations shown in Figure 5, where the external background noise was strong for those intervals. It is worth noting that detection of a specific transformer is most biased by additional background noise (like other electric devices). In the case of type discrimination (*c*_2_ feature), the vital information can be obtained for frequencies until 25 Hz and near 200 Hz. These frequencies are sufficient to precisely define a transformer type and thus are the most robust to background noise. Finally, in the case of the power of the transformer, the vital information is stored near frequencies of 25, 50, 100, 150, and 200 Hz. This result is consistent with previous results for the frequency range 2–100 Hz [8] and the results found in [1,2,3,10,11]. Finally, in most cases, the frequency interval of 1 Hz is sufficient for precise classification for each feature, which strongly decreases the data usage. It is worth noting that more precision is required near 20 Hz (0.5 Hz). The final results for classification and data reduction using the proposed method are presented in Table 3.

The research presented in [12,13,14,15] proposes methods that precisely detect transformer flaws and discharge with an accuracy above 90%; nevertheless, the sensors have to be placed in close vicinity of a transformer. The proposed method, using low-frequency sound, allows the detection of basic parameters of various transformers from a distance of 50 m with an accuracy of at least 80%. It is worth noting that this type of sound has a strong influence on health [1,2] so this type of simple detection from a distance verifies the parameters of a transformer from a distance. The presented results show that various data sets need to be used depending on the task. The data reduction was achieved by decreasing the frequency resolution up to 1 Hz. An additional reduction is characteristic for a specific feature. In the case of indoor/outdoor transformer detection, only two frequency ranges were vital, thus the achieved reduction is high (98%). In the case of the specific transformer model, it was crucial to analyze a wide frequency range to find dissimilarities, thus the reduction was the smallest and depended on the interval resolution reduction. It is worth noting that the SVM ml MODEL requires bigger data precision (only 50% reduction); however, it obtained the best accuracy. The other classifiers obtained lower results; however, the data reduction was more significant, e.g., kNN obtained an 87% reduction at the cost of an accuracy decrease by 3%. In the case of the transformer type, all classifiers obtained a high 99% accuracy; however, the kNN outperformed the other classifiers in accuracy and data reduction. Finally, the power of a transformer can be precisely detected using kNN, SVM, and the MLP network. In the case of the SVM classifier, the accuracy was higher by 0.7%; however, this was at the cost of a lower data reduction. On the other hand, the MLP and kNN classifier obtained a comparable accuracy and data reduction.

## 5. Conclusions

The paper proposed a method to detect distribution transformer parameters from a distance, without the need of installing a multiple sensor array on the transformer. The proposed method uses genetic algorithms to find the optimum frequency representation for detecting a transformer model, its type, and rated power. In every case, the proposed method allowed an increase of the accuracy by 5% on average while finding the optimum intervals and their resolution and decreasing the input data set from 50% up to 98% depending on the task. The research confirmed that in the case of generated power, its harmonics are based on 50 Hz; however, an important interval can also be found near 20 Hz, which are infrasound signals. The specific transformer model can generate the noise in all of the analyzed spectrum; thus, in the case of all machine learning algorithms, the reduction of the interval caused a decrease in the detection accuracy. Finally, the type of the transformer due to its different characteristics (power level and interval construction) obtained a near 100% accuracy while a significant data reduction (over 90%) was achieved. The model, with the SVM classifier, can be applied for solutions requiring maximum accuracy, while models based on kNN and MLP can be applied in edge sensors, due to the significant data reduction.

Further research will be focused on anomaly detection during the operation of transformers based on various characteristics. This method will allow analysis of the technical condition of the transformer based on the measurement of its low-frequency signals made online (without switching off).

## Figures and Tables

**Table 1:** List of the transformers under study.

Transformer Number	Manufacturer	Apparent Power	Transformer Type	Cooling Type
Transformer 1	Schneider Electric	2500 kVA	indoor type	dry-type
Transformer 2	Schneider Electric	2000 kVA	indoor type	dry-type
Transformer 3	Schneider Electric	1600 kVA	indoor type	dry-type
Transformer 4	Schneider Electric	1250 kVA	overhead type	oil-type
Transformer 5	Schneider Electric	630 kVA	overhead type	oil-type
Transformer 6	ABB	400 kVA	overhead type	oil-type
Transformer 7	ABB	400 kVA	overhead type	oil-type
Transformer 8	ABB	250 kVA	overhead type	oil-type
Transformer 9	ABB	250 kVA	overhead type	oil-type
Transformer 10	ABB	250 kVA	overhead type	oil-type
Transformer 11	ABB	250 kVA	overhead type	oil-type
Transformer 12	ABB	250 kVA	overhead type	oil-type
Transformer 13	ABB	160 kVA	overhead type	oil-type
Transformer 14	ABB	100 kVA	overhead type	oil-type
Transformer 15	ABB	100 kVA	overhead type	oil-type
Transformer 16	ABB	63 kVA	overhead type	oil-type

**Table 2:** The result of finding background representation.

	Cross-Validation Error [%]		
Error Rate for Number of Vectors (vmax)	Specific Transformer (*c*_1_),	Transformer Type (*c*_2_)	Transformer Power (*c*_3_)
No background	23.3	0.4	18
1	23.1	0.4	16.1
2	22.8	0.4	15.9
3	23	0.5	17.22
4	24	0.6	18

**Table 3:** Results of the classification for a specific algorithm.

Feature	Algorithm	Accuracy (Based)	Accuracy (Proposed Method)	Data Reduction (Proposed Method)
**Transformer model (*c*_1_)**	kNN	77%	81%	87%
	Bayes	70%	79%	70%
	Random Forest	68%	75%	91%
	SVM	83%	84%	11%
	MLP	74%	77%	81%
**Transformer type (*c*_2_)**	kNN	98.9%	99.99%	98%
	Bayes	97%	99.99%	98.5%
	Random Forest	98.8%	99.8%	97.7%
	SVM	99.8%	99.99%	97.8%
	MLP	99.2%	99.98%	97.1%
**Transformer Power (*c*_3_)**	kNN	78%	87%	93%
	Bayes	73%	78%	93%
	Random Forest	74%	79.1%	95%
	SVM	85%	87.7%	50%
	MLP	79%	86.8%	92%
